# Trends and factors associated with liver fibrosis-cirrhosis complicated with pneumonia-influenza mortality in U.S. Counties: a CDC WONDER and CHR database analysis

**DOI:** 10.3389/fpubh.2026.1884089

**Published:** 2026-08-12

**Authors:** Yanqiu Zhang, Yueming Shen

**Affiliations:** Department of Digestive Diseases, The Affiliated Changsha Central Hospital, Hengyang Medical School, University of South China, Changsha City, China

**Keywords:** core risk factors, liver fibrosis-cirrhosis complicated with pneumonia-influenza, mortality trend, risk phenotype, spatial heterogeneity

## Abstract

**Background:**

The disease burden of liver fibrosis-cirrhosis complicated with pneumonia-influenza has become a major public health concern in the world, yet the long-term trends, and core influencing factors remain unclear. This study aimed to systematically characterize the ecological associations between U.S. county-level health-related risk phenotypes and comorbidity mortality, and to identify area-level factors associated with mortality variation.

**Methods:**

Data were integrated from the Centers for Disease Control and Prevention Wide-ranging Online Data for Epidemiologic Research (CDC WONDER) and 2020 County Health Rankings (CHR) databases. Joinpoint regression was used to examine long-term mortality trends from 1999 to 2023. Unsupervised clustering (PCA combined with HCPC) was then applied to classify county-level phenotypic profiles. Spatial autocorrelation analysis (global and local Moran’s I) was performed to evaluate geographic clustering, and generalized linear models were constructed to assess phenotypic associations with mortality and to identify key correlated health behavior factors.

**Results:**

A total of 435 U.S. counties with complete mortality data from 2018 to 2023 were enrolled, with a total of 32,093 deaths recorded from this comorbidity during the study period. County-level comorbidity mortality showed a phased increase from 2011 to 2023 (Annual Percentage Change [APC] = 6.28%). Three heterogeneous county-level ecological phenotypes were identified via unsupervised clustering. No significant spatial autocorrelation was detected for county-level comorbidity mortality (global Moran’s I = −0.0003, *p* = 0.317), indicating a random spatial distribution without geographic clustering. Phenotype 2, geographically concentrated in the Appalachian and southeastern states, exhibited the highest county-level comorbidity mortality (27.8 per 1,000,000 population) and showed an independent statistical association with elevated mortality relative to other phenotypes (adjusted rate ratio [RR] = 1.304). Furthermore, the county-level average number of physically unhealthy days, disconnected youth rate, and adults with obesity rate were not only strongly associated with high mortality in Phenotype 2 but also positively correlated with county-level single-disease mortality.

**Conclusion:**

County-level comorbidity mortality from this condition showed a phased upward trend with acceleration after 2011 and significant phenotypic specificity. The observed ecological associations between high-risk phenotypes and correlated area-level indicators provide observational reference for geographically tailored public health strategies.

## 1 Introduction

Patients with liver fibrosis-cirrhosis are susceptible to various pathogens due to impaired immune function, and once infected with influenza or pneumonia, their clinical manifestations are more severe and mortality is higher, suggesting that the coexistence of the two may have a synergistic damage effect on disease progression (1–3). Previous studies have confirmed that influenza virus infection can induce Acute-on-Chronic-Liver-Failure (ACLF) (1). In the intensive care unit for liver diseases, the mortality rate of patients with liver cirrhosis infected with the influenza virus was as high as 81.8%, and secondary (fungal or bacterial) pulmonary infections were identified as independent risk factors for the severity of the disease (2). Beyond these hospital-based clinical observations, nationwide population datasets further underscore the national public health burden of this comorbidity. The study based on the National Inpatient Sample (NIS) in the United States also demonstrated that patients with cirrhosis who contracted COVID-19 exhibited higher mortality risks and complication rates compared to influenza patients (3). Moreover, propensity-score matched analyses of the U.S. NIS covering millions of hospital admissions demonstrated that cirrhosis conferred a more than two-fold increased risk of in-hospital death among patients hospitalized for influenza, alongside elevated sepsis and prolonged hospitalization costs (4). Similarly, a nationwide analysis of the 2016–2022 NIS database revealed that among over 4.7 million hospitalized cirrhosis patients, those with pneumococcal pneumonia had substantially higher odds of in-hospital mortality (aOR 2.95), intensive care unit admission (aOR 7.55), and longer hospital stays (5). Collectively, clinical single-center evidence and national inpatient cohorts all demonstrate that coexisting chronic liver disease and severe respiratory infections drastically amplify the risk of fatal adverse outcomes, establishing an urgent need for systematic population-based research characterizing the long-term spatiotemporal burden and ecological drivers of this synergistic comorbidity.

However, these clinical observations are mostly derived from single-center hospital cohorts or national inpatient database analyses focusing on individual-level patient outcomes. Existing population-based county-level epidemiological studies only separately investigate mortality disparities of cirrhosis or pneumonia-influenza as independent endpoints, failing to quantify the population-level excess mortality burden caused by their synergistic comorbidity (6–8). Moreover, prior investigations have relied primarily on conventional regression models or predefined stratifications (e.g., by state, urban–rural status, or race/ethnicity), which depend on *a priori* assumptions and may miss non-linear, multidimensional risk configurations. By contrast, unsupervised phenotypic clustering has proven effective in cardiovascular research for identifying county-level phenogroups with distinct risk profiles that traditional methods fail to detect, yet this approach has not been applied to liver disease-respiratory infection comorbidity (9). Third, no existing study has integrated long-term mortality trend analysis with cross-sectional ecological association modeling within a unified framework for this comorbidity, limiting the ability to simultaneously assess temporal evolution and current risk correlates.

To address the aforementioned research gaps, this study proceeded in two steps. First, using Centers for Disease Control and Prevention Wide-ranging Online Data for Epidemiologic Research (CDC WONDER) data from 1999 to 2023, we analyzed the long-term trends in county-level mortality for liver fibrosis-cirrhosis complicated with pneumonia-influenza. Second, integrating the 2020 County Health Rankings (CHR) database for county-level health indicators, we applied unsupervised clustering to identify distinct county-level health phenotypes, and further investigated the ecological associations between phenotype groups and comorbidity mortality, as well as the key county-level health behavior factors linked to mortality. This work provides an ecological evidence base for designing spatially targeted and population-specific public health interventions and highlights the need for further individual-level investigations to validate these area-level findings.

## 2 Methods

### 2.1 Study setting and population

**Figure 1:**
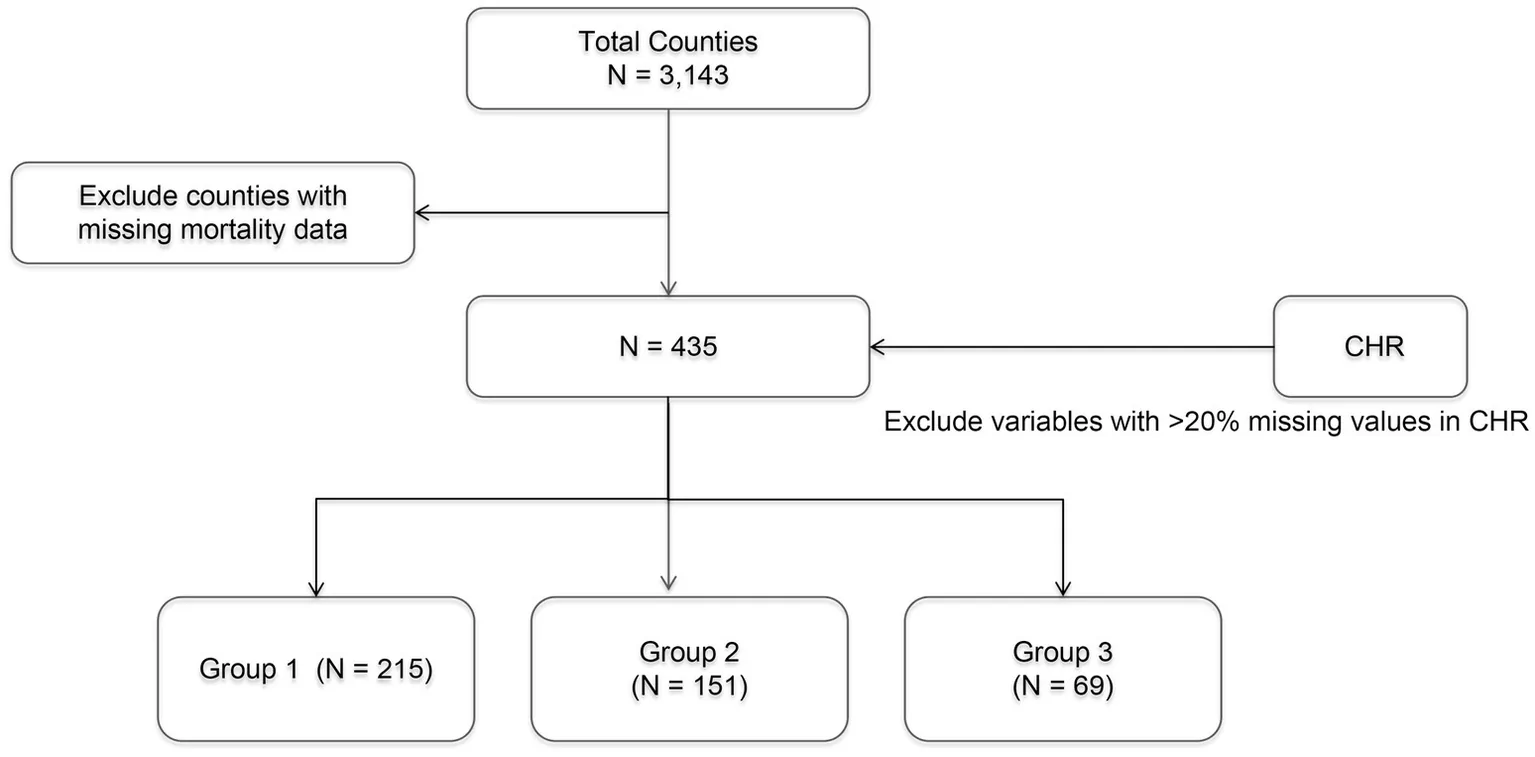
The workflow of this study.

To systematically assess the social, environmental, behavioral, and demographic factors associated with liver fibrosis-cirrhosis complicated with pneumonia-influenza at the county level, as well as associated spatial distribution and long-term trends, this study adopted an ecological analysis design at the U.S. county level. The CDC WONDER database was chosen for its nationwide coverage, continuous 24-year time series, and county-level geographic availability, enabling robust trend and spatial analyses. The 2020 County Health Rankings (CHR) was selected as the exposure data source because it provides standardized, multi-dimensional county-level measures across health behaviors, clinical care, socioeconomic factors, and physical environment, and is publicly accessible and reproducible. For phenotypic classification, we adopted an unsupervised clustering approach (PCA-HCPC) following the methodological framework of Segar et al., which has been applied to county-level health phenomapping in cardiovascular research (9). 435 U.S. counties with complete disease mortality data from 2018 to 2023 were designated as research units. The workflow of this study was shown in Figure 1.

### 2.2 Data abstraction

To clarify data sources, this study integrated two core databases. First, the 1999–2023 CDC WONDER (https://wonder.cdc.gov/) was compiled by 57 vital statistics jurisdictions, and it includes variables like residence, age group, race, gender, year and urbanization level of U.S. county residents. It also supports extracting underlying causes of death via 4-digit International Classification of Diseases (ICD) codes, enables mortality calculation per 1,000,000 population with 95% confidence intervals (CI) and standard errors provided, and facilitates extracting the 2018–2023 average annual crude mortality rate of liver fibrosis-cirrhosis (ICD-10 K74.1-K74.6) complicated with pneumonia-influenza (ICD-10 J09-J18 plus subcategories) as the outcome indicator. Specifically, a comorbidity death was defined as a death certificate in which ICD-10 codes for liver fibrosis-cirrhosis (K74.1-K74.6) and for pneumonia-influenza (J09-J18 and subcategories) both appeared in any field—either as the underlying cause of death or as any of the multiple contributing causes. For single-disease analyses, the CDC WONDER Multiple Cause of Death (MCOD) database was used, where a death was classified as related to a specific disease if the target ICD-10 code appeared in either the underlying cause of death or any of the multiple cause fields. The second database is the 2020 County Health Rankings (CHR, https://www.countyhealthrankings.org/), which integrates multi-source data across 4 dimensions (health behavior, clinical care, socioeconomic factors and physical environment) for over 3,000 U.S. counties (9). For preprocessing, counties with more than 20% missing covariates were excluded, and 68 candidate variables were evaluated via Pearson correlation coefficients: those with a correlation coefficient greater than 0.7 were removed, while upstream predictive variables in correlated groups were retained. Additionally, continuous variables were standardized to a mean of 0 and a standard deviation of 1, and missing values were imputed using multiple imputation by chained equations, ultimately resulting in the inclusion of 43 variables with no missing rate exceeding 20%.

### 2.3 Statistical analysis

All analyses were conducted in R software (v4.5.1) with statistical significance set at a 2-tailed *p* < 0.05.

To identify long-term evolutionary characteristics of U.S. mortality from liver fibrosis-cirrhosis complicated with pneumonia-influenza (1999–2023), Joinpoint regression was used to fit piecewise linear models, locate trend turning points, and calculate annual percentage changes via R package segmented (v2.1.4) (10). The number of joinpoints was determined by systematically comparing models with 0, 1, and 2 joinpoints, using the Bayesian Information Criterion (BIC) as the primary selection criterion, supplemented by the Davies test to formally assess the presence of at least one joinpoint.

Data redundancy was first eliminated by Principal Component Analysis (PCA) for dimensionality reduction, followed by Hierarchical Clustering on Principal Components (HCPC), using the R package FactoMineR (v2.12). This hybrid method combines agglomerative hierarchical clustering with K-means clustering. The optimal number of phenotypes was determined through a multi-criteria approach: the inertia gain criterion from the hierarchical clustering tree, the elbow plot of within-cluster sum of squares, and silhouette analysis across candidate cluster solutions (k = 2 to 8). To assess clustering stability, bootstrap resampling (100 iterations) was performed, and the adjusted Rand index (ARI) was calculated for the selected cluster solution. V statistics were calculated to identify variable over−/under-representation across phenotypes. Baseline continuous variables were presented as medians with interquartile ranges (IQR), and one-way analysis of variance was used to test differences among phenotypic groups.

To evaluate spatial clustering of county-level comorbidity mortality, spatial weight matrices were constructed by the k-nearest neighbor (k = 5) algorithm based on the true geographic centroids of U.S. counties obtained from the U.S. Census Bureau via the R package usmap (v1.0.0), and global/local Moran’s I indices were applied, with Local Indicators of Spatial Association (LISA) hotspot maps generated. One-way analysis of variance was used to test mortality differences across spatial clusters (hotspots, coldspots, and non-significant areas).

Least Absolute Shrinkage and Selection Operator (LASSO) regression was utilized to screen candidate clinical variables (10-fold cross-validation for optimal parameters) via the R package glmnet (v4.1–8), ranking variables by coefficient importance and selecting predictors. A Gamma generalized linear model (log link, Phenotype1 as reference) was constructed, including unadjusted and adjusted models (adjusted for selected variables and age), with associations between phenotypes and mortality reported as relative risk with 95% confidence interval. V statistics analyzed differential characteristics of the three county phenotypes. Additionally, for the three most prominent characteristic variables identified by V statistics in the phenotype with highest mortality, separate generalized linear models were built for comorbidity mortality and single-disease mortality (hepatitis-related cirrhosis and pneumonia-influenza), adjusting for socioeconomic and environmental confounders including proportion of population aged ≥ 65 years, income ratio, female proportion, low birth weight rate, PM₂.₅ concentration, excessive drinking rate, housing cost burden, and high school graduation rate, to examine the association patterns of these key variables across outcomes.

## 3 Results

### 3.1 County-level sample characteristics and representativeness

Among the 3,142 U.S. counties, 435 (13.8%) met the inclusion criteria of complete mortality data from 2018 to 2023 and < 20% missing covariates. A comparison of demographic and health characteristics between included and excluded counties (Supplementary Table 1) revealed significant differences across multiple socioeconomic and health indicators: included counties had substantially larger populations, higher urbanization levels, and greater healthcare resource availability, whereas excluded counties were more likely to be rural, with higher poverty rates and more limited healthcare access. This pattern suggests that missingness was not completely random, but rather reflected systematic selection bias driven by uneven surveillance system coverage and CHR data availability.

### 3.2 The comorbidity mortality trends from 1999 to 2023 at the county level

**Figure 2:**
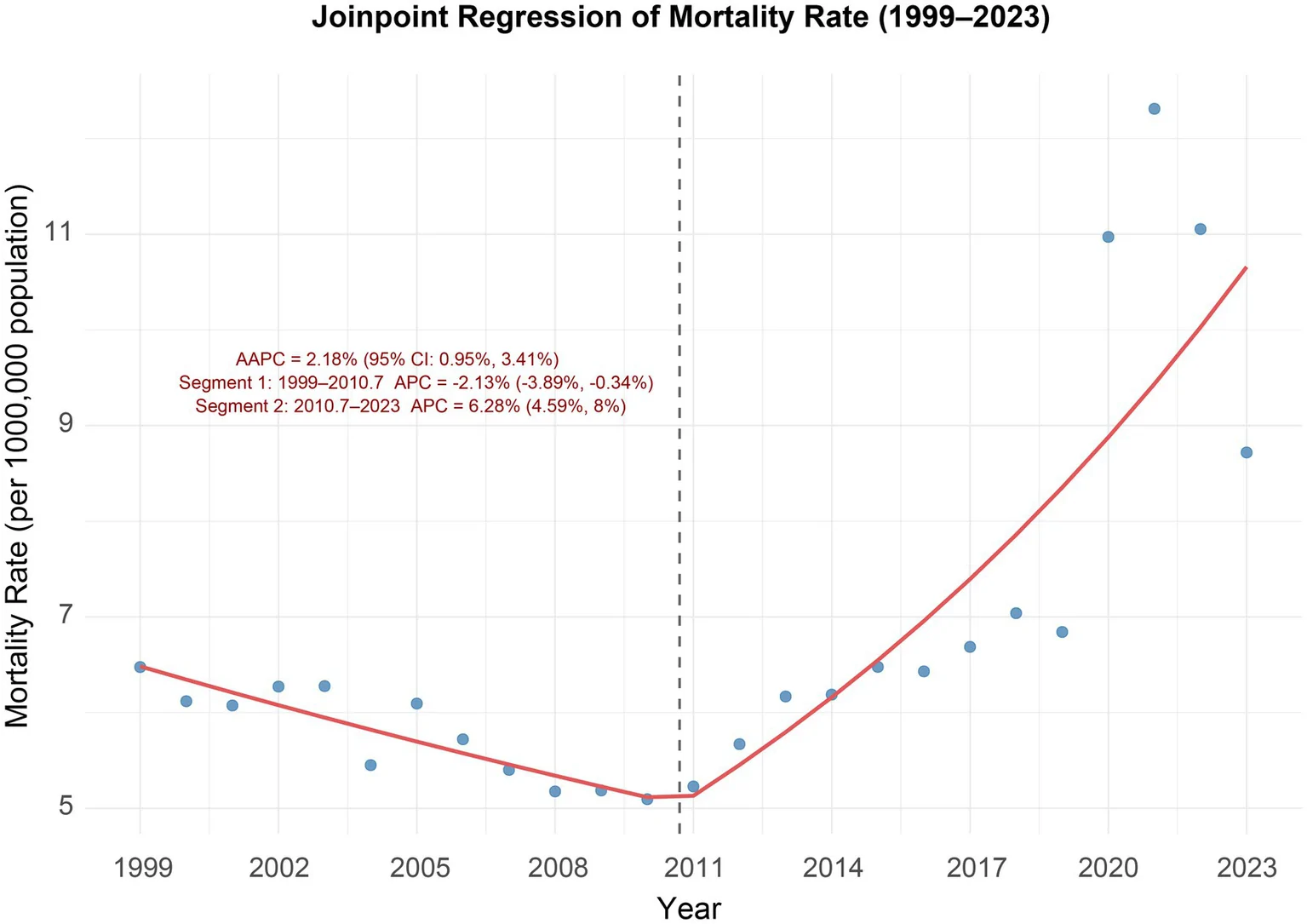
Mortality trends of liver fibrosis-cirrhosis complicated with pneumonia-influenza from 1999 to 2023.

Joinpoint regression identified phased changes in U.S. county-level mortality from liver fibrosis-cirrhosis complicated with pneumonia-influenza over the 24-year period. The optimal number of joinpoints was determined by systematically comparing models with 0, 1, and 2 joinpoints. The Davies test showed *p* < 0.0001, strongly rejecting the null hypothesis of no joinpoint. BIC values were −5.3 for the linear model (0 joinpoints), −27.5 for the 1-joinpoint model, and −27.0 for the 2-joinpoint model; the 1-joinpoint model had the lowest BIC and did not meaningfully improve with an additional parameter, and was therefore selected as the optimal model. Over the period from 1999 to 2011, the average annual percentage change (APC) in mortality was −2.13% (95% CI: −3.89 to −0.34%), demonstrating a significant downward trend (Figure 2). From 2011 to 2023, the APC shifted to 6.28% (95% CI: 4.59 to 8.00%), indicating a significant upward trend. For the entire study period (1999–2023), the average annual percentage change (AAPC) was 2.18% (95% CI: 0.95 to 3.41%).

### 3.3 County-level phenotype classification and baseline characteristics

**Figure 3:**
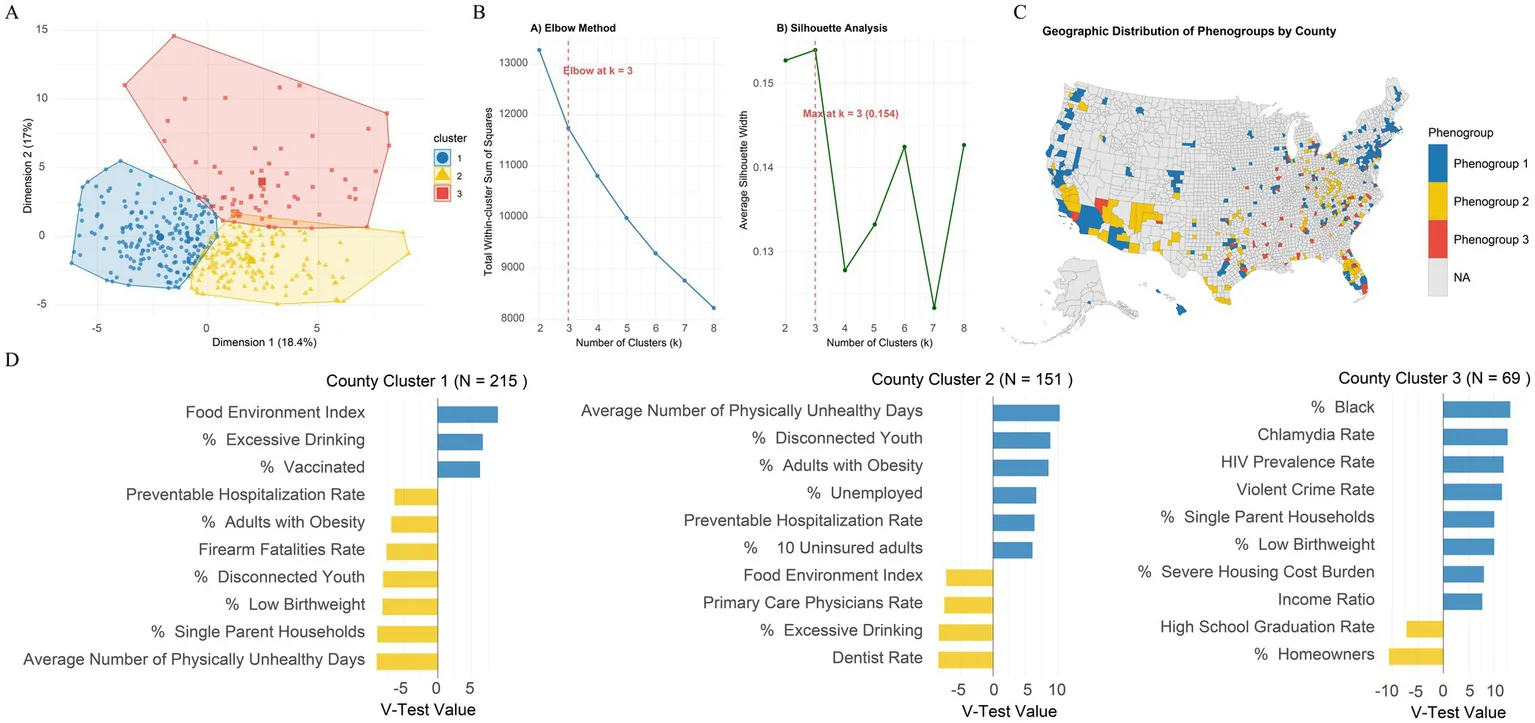
Phenotype classification at the county level **(A)** The 2-dimensional distribution of the three phenotypes. **(B)** Clustering stability assessment. **(C)** The geographic distribution of U.S. county-level phenotypes. **(D)** V-test results of characteristic indicators for county phenotypes.

Unsupervised clustering (PCA combined with HCPC) identified three distinct county-level phenotypes with minimal overlap and stable cluster structures (Figure 3A). The robustness of the 3-cluster solution was supported by multiple quantitative metrics: silhouette analysis yielded an average silhouette width of 0.154 (ranging from 0.062 for Phenotype 2 to 0.231 for Phenotype 1), and the elbow plot of within-cluster sum of squares showed a clear inflection point at k = 3. Bootstrap resampling (100 iterations) produced a mean adjusted Rand index (ARI) of 0.267 (median 0.254) for the 3-cluster assignment, indicating acceptable clustering stability (Figure 3B). Phenotype 1 concentrated in western and northeastern coastal areas, Phenotype 2 concentrated in the Appalachian and southeastern states (including Texas, Florida, North Carolina, Kentucky, and West Virginia), and Phenotype 3 scattered in the east (Figure 3C).

Distinct baseline characteristics were delineated across different phenotypes, with significant variations in most health behavior, clinical care, socioeconomic and physical environment indicators, and each group displaying a unique combination of health social determinants linked to comorbidity mortality risk variations, showing notable heterogeneity among the 3 clusters from 435 counties (Table 1). Specifically, Phenotype 2 had the highest values in average number of physically unhealthy days (4.3 days), adults with obesity rate (34.0%), preventable hospitalization rate (5360.0) and disconnected youth rate (9.0%), plus the lowest primary care physician rate (53.0) and low dentist rate. Phenotype 3 recorded the highest chlamydia rate (820.8) and income ratio (5.1), the worst food environment index (6.9), as well as a high proportion of Black population, high violent crime rate, low homeowners rate and low high school graduation rate. Phenotype 1 had the optimal food environment index (8.1), alongside a high proportion of excessive drinking, and low average number of physically unhealthy days. Only a few indicators (e.g., proportion of less than 18 years of age) showed no inter-group differences. V statistics further confirmed distinct group profiles (Figure 3D), consistent with the above traits.

**Table 1:** Baseline characteristics comparison of phenotypes.

Variable	Overall	Phenotype1	Phenotype2	Phenotype3	*p*
	435	215	151	69	
Average number of physically unhealthy Days	3.9 (3.5, 4.3)	3.5 (3.2, 3.9)	4.3 (4.1, 4.8)	4.0 (3.7, 4.3)	<0.001
% low birthweight	8.0 (7.0, 9.0)	8.0 (7.0, 8.0)	9.0 (8.0, 9.0)	10.0 (9.0, 11.0)	<0.001
% adults with obesity	31.0 (27.0, 35.0)	29.0 (25.0, 32.0)	34.0 (32.0, 37.0)	31.0 (27.0, 35.0)	<0.001
Food environment index	7.7 (7.0, 8.2)	8.1 (7.7, 8.6)	7.1 (6.8, 7.6)	6.9 (6.6, 7.6)	<0.001
% excessive drinking	18.0 (16.0, 20.0)	19.0 (18.0, 21.0)	16.0 (15.0, 18.0)	18.0 (16.0, 20.0)	<0.001
% driving deaths with alcohol involvement	29.0 (24.0, 33.0)	29.0 (25.0, 33.0)	27.0 (22.0, 32.0)	28.0 (19.0, 34.0)	0.006
Chlamydia rate	463.8 (332.3, 629.3)	401.0 (322.0, 528.3)	457.5 (309.4, 590.3)	820.8 (706.1, 1,066.2)	<0.001
Primary care physicians rate	70.0 (53.0, 90.0)	77.0 (62.0, 96.0)	53.0 (43.0, 67.0)	88.0 (75.0, 108.0)	<0.001
Dentist rate	64.0 (49.0, 77.0)	71.0 (57.0, 83.0)	49.0 (37.0, 59.0)	75.0 (68.0, 88.0)	<0.001
Mental health provider rate	206.0 (126.0, 322.0)	245.0 (152.0, 343.0)	149.0 (89.0, 223.0)	289.0 (208.0, 373.0)	<0.001
Preventable hospitalization rate	4,721.0 (3,861.0, 5,520.0)	4,179.0 (3,379.0, 4,800.0)	5,360.0 (4,561.0, 6,414.0)	5,194.0 (4,513.0, 5,894.0)	<0.001
% with annual mammogram	42.0 (38.0, 46.0)	43.0 (40.0, 47.0)	39.0 (35.0, 43.0)	42.0 (37.0, 45.0)	<0.001
% vaccinated	48.0 (43.0, 51.0)	50.0 (47.0, 53.0)	44.0 (39.0, 49.0)	46.0 (41.0, 50.0)	<0.001
High school graduation rate	88.0 (83.0, 91.0)	89.0 (85.0, 92.0)	88.0 (82.0, 91.0)	82.0 (77.0, 85.0)	<0.001
% unemployed	3.8 (3.3, 4.7)	3.5 (3.0, 4.0)	4.5 (3.7, 5.9)	4.0 (3.6, 4.9)	<0.001
income ratio	4.6 (4.2, 5.0)	4.4 (4.1, 4.7)	4.6 (4.4, 5.1)	5.1 (4.8, 5.6)	<0.001
% single-parent households	34.0 (29.0, 39.0)	29.0 (24.0, 33.0)	37.0 (33.0, 40.0)	42.0 (39.0, 48.0)	<0.001
Social association rate	9.0 (7.1, 11.2)	8.6 (7.0, 10.4)	9.6 (6.4, 11.7)	9.9 (8.1, 11.7)	0.009
Violent crime rate	336.0 (215.0, 476.0)	263.0 (173.0, 373.0)	333.0 (221.0, 453.0)	615.0 (532.0, 815.0)	<0.001
Average daily PM2 5	10.0 (8.8, 10.9)	10.0 (8.5, 10.8)	9.9 (8.6, 10.8)	10.3 (9.5, 11.5)	0.030
% drive alone to work	81.0 (78.0, 83.0)	80.0 (76.0, 83.0)	82.0 (80.0, 85.0)	80.0 (72.0, 83.0)	<0.001
% long commute––drives alone	33.0 (25.0, 42.0)	37.0 (29.0, 44.0)	31.0 (24.0, 36.0)	31.0 (24.0, 44.0)	<0.001
HIV prevalence rate	212.0 (139.0, 331.0)	191.0 (135.0, 281.0)	175.0 (124.0, 249.0)	602.0 (431.0, 868.0)	<0.001
% insufficient sleep	35.0 (32.0, 37.0)	33.0 (31.0, 35.0)	35.0 (33.0, 37.0)	37.0 (35.0, 39.0)	<0.001
% uninsured adults	12.0 (8.0, 16.0)	9.0 (7.0, 14.0)	15.0 (10.0, 19.0)	13.0 (9.0, 16.0)	<0.001
Other primary care provider rate	93.0 (69.0, 133.0)	87.0 (64.0, 120.0)	86.0 (69.0, 117.0)	143.0 (106.0, 183.0)	<0.001
% disconnected youth	7.0 (5.0, 9.0)	5.0 (4.0, 7.0)	9.0 (7.0, 11.0)	7.0 (6.0, 9.0)	<0.001
Segregation index (residential segregation–Black/White)	50.0 (43.0, 58.0)	49.0 (42.0, 56.0)	50.0 (42.0, 59.0)	56.0 (48.0, 66.0)	<0.001
Segregation index (residential segregation–non-white/white)	36.0 (29.0, 45.0)	34.0 (29.0, 41.0)	33.0 (26.0, 43.0)	47.0 (38.0, 55.0)	<0.001
Firearm fatalities rate	13.0 (9.0, 16.0)	10.0 (7.0, 13.0)	16.0 (13.0, 18.0)	16.0 (11.0, 20.0)	<0.001
Juvenile arrest rate	21.0 (14.0, 29.0)	18.7 (12.0, 26.0)	22.7 (18.0, 33.0)	23.7 (15.0, 29.1)	<0.001
Average traffic volume per meter of major roadways	212.0 (102.0, 401.0)	266.0 (151.0, 490.0)	107.0 (62.0, 197.0)	465.0 (285.0, 871.0)	<0.001
% Homeowners	67.0 (61.0, 72.0)	67.0 (63.0, 73.0)	69.0 (65.0, 73.0)	55.0 (50.0, 61.0)	<0.001
% severe housing cost burden	14.0 (11.0, 16.0)	14.0 (11.0, 16.0)	12.0 (11.0, 14.0)	17.0 (15.0, 19.0)	<0.001
Population y	291,538.0 (151,096.0, 596,849.0)	415,247.0 (219,564.0, 705,388.0)	132,064.0 (70,503.0, 222,846.0)	678,701.0 (323,780.0, 1,050,114.0)	<0.001
% less than 18 years of age	22.2 (20.7, 23.9)	22.1 (20.8, 23.9)	22.2 (20.5, 24.3)	22.7 (20.9, 23.6)	0.930
% 65 and over	15.9 (13.8, 18.3)	15.7 (13.8, 17.8)	17.9 (14.9, 20.4)	14.1 (12.8, 16.0)	<0.001
% Black	7.6 (3.1, 15.9)	6.6 (3.1, 11.0)	5.5 (1.9, 11.2)	29.2 (20.0, 40.8)	<0.001
% American Indian and Alaska native	0.6 (0.4, 1.1)	0.7 (0.4, 1.1)	0.6 (0.4, 1.3)	0.5 (0.4, 0.9)	0.321
% Asian	2.6 (1.3, 4.9)	3.9 (2.2, 6.4)	1.2 (0.7, 1.9)	3.5 (2.1, 5.8)	<0.001
% native Hawaiian/Other Pacific Islander	0.1 (0.1, 0.2)	0.1 (0.1, 0.2)	0.1 (0.1, 0.2)	0.1 (0.1, 0.2)	0.005
% non-Hispanic White	68.0 (51.5, 80.5)	70.8 (59.3, 80.6)	72.2 (55.6, 87.4)	44.9 (35.6, 56.7)	<0.001
% Female	51.0 (50.4, 51.5)	50.8 (50.3, 51.2)	51.0 (50.3, 51.4)	51.7 (51.5, 52.5)	<0.001

Data are presented as median (interquartile range, IQR) for continuous variables.

### 3.4 The spatial heterogeneity of comorbidity mortality at the county level

No significant spatial autocorrelation was detected for county-level comorbidity mortality (global Moran’s I = −0.0003, *p* = 0.317). Local LISA analysis identified no significant hotspots or coldspots (all 435 counties were non-significant). These results indicate that the county-level mortality from liver fibrosis-cirrhosis complicated with pneumonia-influenza is randomly distributed across the U.S., without evidence of geographic clustering.

### 3.5 Associations between phenotypes and comorbidity mortality at the county level

During 2018–2023, a total of 32,093 deaths from liver fibrosis-cirrhosis complicated with pneumonia-influenza were recorded across the 435 counties included in this study. Phenotypes differed significantly in comorbidity mortality rates. Phenotype 2 had the highest mortality rate at 27.8/1,000,000 population (95% CI: 24.3–31.3), while Phenotypes 1 and 3 showed comparable rates at 9.4/1,000,000 (95% CI: 8.8–9.9) and 9.8/1,000,000 (95% CI: 8.6–10.9), respectively, with no confidence interval overlap (Table 2). LASSO regression further identified the phenotype group along with nine covariates as important predictors for comorbidity mortality: average number of physically unhealthy days, disconnected youth rate, income ratio, female population proportion, low birth weight rate, average daily PM2.5 concentration, excessive drinking rate, proportion with severe housing cost burden, and high school graduation rate. To examine mortality differences across phenotypes, a Gamma generalized linear model with a log link was constructed, adjusting for the nine LASSO-selected covariates plus the proportion of the population aged 65 years and older. In the crude model, Phenotype 2 showed significantly higher mortality compared with Phenotype 1 (RR = 2.972, 95% CI: 2.625–3.366, *p* < 0.001), while Phenotype 3 did not differ significantly. After adjustment for confounders, Phenotype 2 remained associated with significantly higher mortality than Phenotype 1 (RR = 1.304, 95% CI: 1.159–1.466, p < 0.001) (Table 3). The marked reduction in the rate ratio from 2.972 to 1.304 indicates that a substantial portion of the crude mortality gradient was explained by the included covariates—particularly the higher burden of physically unhealthy days, disconnected youth, obesity, and lower healthcare access characterizing Phenotype 2. Nonetheless, the residual excess risk (RR = 1.304) suggests that the phenotype classification captures additional risk beyond these measured covariates, possibly reflecting unmeasured factors such as healthcare quality, health literacy, or vaccination coverage, or synergistic effects of the risk factor clustering pattern itself.

**Table 2:** Mortality rate comparison across phenotypes.

Variable	Phenotype 1 (95% CI)	Phenotype 2 (95% CI)	Phenotype 3 (95% CI)
Mortality rate	9.4 (8.8–9.9)	27.8 (24.3–31.3)	9.8 (8.6–10.9)

95% CI: 95% confidence interval.

**Table 3:** Crude and adjusted model rate ratios of phenotypes.

Variable	Model	Estimate	Std. error	Wald *z*	*p*-value	Rate ratio	95% CI
Phenotype1							
Phenotype2	Crude	1.089	0.064	17.125	<0.001	2.972	(2.625, 3.366)
Phenotype3	Crude	0.041	0.083	0.492	0.623	1.042	(0.886, 1.225)
Phenotype1							
Phenotype2	Adjusted	0.265	0.06	4.42	<0.001	1.304	(1.159, 1.466)
Phenotype3	Adjusted	−0.036	0.08	−0.45	0.65	0.964	(0.824, 1.129)

95% CI: 95% confidence interval.

### 3.6 The health behavior factors associated with comorbidity mortality at the county level

According to the V statistics results, average number of physically unhealthy days, disconnected youth rate, and adults with obesity rate were three most prominent characteristic variables of Phenotype 2. To investigate the potential determinants of comorbidity mortality, we further conducted generalized linear model analyses on these three characteristic variables. Results demonstrated that in the overall sample, the average number of physically unhealthy days (adjusted RR = 1.791, 95% CI: 1.634–1.962), disconnected youth rate (adjusted RR = 1.047, 95% CI: 1.029–1.065), and adults with obesity rate (adjusted RR = 1.015, 95% CI: 1.004–1.027) were each independently and positively associated with comorbidity mortality (Table 4). However, stratified analyses by phenotype revealed differential patterns: the average number of physically unhealthy days remained a robust and independent predictor across all three phenotypes (adjusted RRs ranging from 1.515 to 1.604, all *p* < 0.01), indicating its role as a cross-phenotype core driver. In contrast, the associations of disconnected youth rate and adults with obesity rate were attenuated and no longer statistically significant in Phenotypes 2 and 3, with their independent effects only detectable in the low-mortality Phenotype 1. This pattern suggests that in counties with a high burden of poor physical health (Phenotypes 2 and 3), the contributions of youth disconnection and obesity to mortality may be captured by the more proximal composite measure of physically unhealthy days; conversely, in counties with better overall health status (Phenotype 1), the independent hazards of these social and metabolic factors become discernible. Collectively, the average number of physically unhealthy days emerged as the most consistent determinant of comorbidity mortality across phenotypes, while the independent effects of disconnected youth and obesity were highly dependent on baseline county health levels.

**Table 4:** Crude and adjusted model RRs of three health behavior factors.

Phenogroup	Variable	Model	*N*	Estimate	Std. error	Wald *z*	*p*-value	Rate ratio	95%CI
Overall	Average number of physically unhealthy days	Crude	434	0.809	0.042	19.301	<0.001	2.246	(2.069, 2.438)
Overall	Average number of physically unhealthy days	Adjusted	434	0.583	0.047	12.492	<0.001	1.791	(1.634, 1.962)
Overall	% disconnected youth	Crude	434	0.153	0.010	15.695	<0.001	1.166	(1.144, 1.188)
Overall	% disconnected youth	Adjusted	434	0.046	0.009	5.273	<0.001	1.047	(1.029, 1.065)
Overall	% adults with obesity	Crude	434	0.087	0.006	14.254	<0.001	1.091	(1.078, 1.104)
Overall	% adults with obesity	Adjusted	434	0.015	0.006	2.552	0.011	1.015	(1.004, 1.027)
Phenotype1	Average number of physically unhealthy days	Crude	214	0.525	0.067	7.867	<0.001	1.691	(1.483, 1.927)
Phenotype1	Average number of physically unhealthy days	Adjusted	214	0.472	0.066	7.128	<0.001	1.604	(1.408, 1.826)
Phenotype1	% disconnected youth	Crude	214	0.065	0.018	3.567	<0.001	1.067	(1.030, 1.106)
Phenotype1	% disconnected youth	Adjusted	214	0.073	0.017	4.333	<0.001	1.076	(1.041, 1.113)
Phenotype1	% adults with obesity	Crude	214	0.042	0.006	6.704	<0.001	1.043	(1.030, 1.056)
Phenotype1	% adults with obesity	Adjusted	214	0.022	0.008	2.684	0.007	1.022	(1.006, 1.039)
Phenotype2	Average number of physically unhealthy days	Crude	151	0.675	0.082	8.186	<0.001	1.964	(1.671, 2.309)
Phenotype2	Average number of physically unhealthy days	Adjusted	151	0.459	0.096	4.789	<0.001	1.583	(1.312, 1.910)
Phenotype2	% disconnected youth	Crude	151	0.107	0.015	7.335	<0.001	1.113	(1.082, 1.145)
Phenotype2	% disconnected youth	Adjusted	151	0.015	0.011	1.324	0.186	1.015	(0.993, 1.038)
Phenotype2	% adults with obesity	Crude	151	0.074	0.014	5.397	<0.001	1.077	(1.048, 1.106)
Phenotype2	% adults with obesity	Adjusted	151	0.001	0.011	0.057	0.955	1.001	(0.980, 1.021)
Phenotype3	Average number of physically unhealthy days	Crude	69	0.519	0.115	4.495	<0.001	1.680	(1.340, 2.107)
Phenotype3	Average number of physically unhealthy days	Adjusted	69	0.416	0.137	3.038	0.002	1.515	(1.159, 1.981)
Phenotype3	% disconnected youth	Crude	69	0.073	0.023	3.118	0.002	1.076	(1.027, 1.126)
Phenotype3	% disconnected youth	Adjusted	69	0.039	0.026	1.498	0.134	1.040	(0.988, 1.095)
Phenotype3	% adults with obesity	Crude	69	0.036	0.010	3.614	<0.001	1.037	(1.017, 1.057)
Phenotype3	% adults with obesity	Adjusted	69	0.011	0.016	0.700	0.484	1.012	(0.980, 1.044)

95% CI: 95% confidence interval.

Additionally, during 2018–2023, a total of 601,875 deaths from hepatitis-related cirrhosis and 2,824,525 deaths from pneumonia-influenza were recorded in the MCOD database. Moreover, these three factors were also positively associated with single-disease mortality from hepatitis-related cirrhosis and pneumonia-influenza after adjustment (all *p* < 0.001, Table 5).

**Table 5:** Relative risk analysis of risk factors for hepatitis-related cirrhosis and pneumonia–influenza.

Variables		Hepatitis-related cirrhosis				Pneumonia and influenza			
	Model	Estimate	RR	95%CI	*p*	Estimate	RR	95%CI	*p*
Physical unhealthy days	Crude	0.4061	1.501	(1.454, 1.549)	<0.001	0.3649	1.44	(1.405, 1.477)	<0.001
Physical unhealthy days	Adjusted	0.2995	1.349	(1.297, 1.403)	<0.001	0.2344	1.264	(1.227, 1.303)	<0.001
% disconnected youth	Crude	0.048	1.049	(1.044, 1.055)	<0.001	0.0362	1.037	(1.033, 1.041)	<0.001
% disconnected youth	Adjusted	0.0216	1.022	(1.017, 1.027)	<0.001	0.0144	1.015	(1.011, 1.018)	<0.001
% adults with obesity	Crude	0.0336	1.034	(1.030, 1.039)	<0.001	0.0307	1.031	(1.028, 1.035)	<0.001
% adults with obesity	Adjusted	0.0173	1.017	(1.013, 1.022)	<0.001	0.0166	1.017	(1.014, 1.020)	<0.001

95% CI: 95% confidence interval. RR: rate ratio.

## 4 Discussion

The comorbidity state of liver fibrosis-cirrhosis combined with pneumonia-influenza is a major global public health challenge. Due to pathological physiological changes such as impaired immune function, malnutrition, and portal hypertension, patients with liver fibrosis-cirrhosis have significantly increased susceptibility to pneumonia-influenza (11), and infection can further trigger severe complications such as hepatic encephalopathy and liver-kidney syndrome, forming a “liver disease-infection” vicious cycle, leading to a significant increase in mortality (12, 13).

This study extends the existing county-level disparity literature in three specific ways. First, by integrating Joinpoint trend analysis with cross-sectional phenotyping within a unified framework, our dual-design approach offers a more comprehensive assessment than prior studies that have examined temporal trends and spatial disparities in isolation, enabling simultaneous characterization of “how mortality has evolved over time” and “what county-level characteristics correlate with current mortality differentials.” Over the 1999–2023 study period, the county-level comorbidity mortality rate exhibited a phased evolutionary pattern: a significant downward trend from 1999 to 2011 (APC = −2.13, 95% CI: −3.89 to −0.34%), followed by a sharp reversal to a significant upward trend from 2011 to 2023 (APC = 6.28, 95% CI: 4.59 to 8.00%), yielding an overall average annual percentage change of 2.18% (95% CI: 0.95 to 3.41%) across the full 24-year period. The observed reversal from a declining to an accelerating upward trend in comorbidity mortality after 2011 may be partially attributable to multiple factors operating during this period. The initial downward phase from 1999 to 2011 coincided with improvements in cirrhosis-related mortality (14), likely reflecting advances in hepatitis C treatment, expanded vaccination coverage for influenza and pneumococcal disease, and broader public health interventions targeting chronic liver disease complications (15). However, the subsequent upward shift beginning in 2011 occurred against a backdrop of increasing population-level chronic disease burden, including rising obesity prevalence (16) and the expanding opioid epidemic (17), both of which have been linked to adverse liver and respiratory outcomes in prior ecological and clinical studies. The 2017–2018 influenza season (18, 19), which was among the most severe in recent decades-, may have further amplified mortality among patients with underlying cirrhosis during this upward phase. More recently, the COVID-19 pandemic introduced substantial excess mortality among individuals with pre-existing liver disease (20), likely contributing to the sustained elevation in comorbidity mortality during 2020–2023. However, because these temporal events overlap in time and our Joinpoint analysis models the entire 1999–2023 series with a single turning point, we cannot statistically disentangle the independent contribution of each period-specific factor. The attribution to specific temporal events therefore remains speculative and requires confirmation in future studies incorporating annual time-stratified or period-excluded analytical approaches.

Second, several previous county-level ecological studies have examined mortality disparities for cirrhosis and respiratory infections separately (6, 7). For example, studies utilizing CDC WONDER data have documented substantial geographic clustering of cirrhosis mortality in the southern and Appalachian regions, attributed to higher prevalence of alcohol use, hepatitis C, and metabolic risk factors (6, 8). Similarly, county-level analyses of influenza and pneumonia mortality have identified elevated rates in counties with lower socioeconomic status, reduced vaccination coverage, and higher chronic disease burden (7). Consistent with these prior observations, the high-risk Phenotype 2 identified in our study geographically overlaps with the traditionally recognized high-burden regions for cirrhosis (Appalachia and the Southeast). Despite this concordance, we provide the first systematic quantification of comorbidity mortality—the synergistic burden when both conditions co-occur—at the US county level. We demonstrate that Phenotype 2 exhibits a mortality rate of 27.8 per 1,000,000 population, which substantially exceeds the rates reported for either condition alone in previous county-level analyses, underscoring that single-disease assessments may significantly underestimate the population-level mortality burden of this comorbidity. Furthermore, the specific configuration of characteristics defining Phenotype 2—particularly the combination of high physically unhealthy days, high disconnected youth, and high obesity alongside low healthcare access—differs from the risk factor profiles typically emphasized in single-disease studies, which have tended to focus on alcohol use, hepatitis prevalence, or vaccination rates. This configurational difference underscores the value of a comorbidity-specific analytical lens for identifying counties with distinct, multifaceted risk profiles that may require integrated intervention approaches.

Third, unlike conventional regression-based disparity analyses that rely on pre-specified stratifications or examine individual risk factors in isolation, our unsupervised clustering framework identifies three data-driven county phenotypes defined by configurations of interconnected characteristics—notably, the high-risk Phenotype 2 is characterized by the simultaneous co-occurrence of elevated physically unhealthy days, high disconnected youth rate, high obesity prevalence, and low healthcare accessibility. This configurational insight, which cannot be obtained from single-variable or stratified regression models, provides a more ecologically valid representation of real-world county-level risk profiles.

The three county-level characteristics most prominently over-represented in this high-mortality phenotype—average number of physically unhealthy days, disconnected youth rate, and adults with obesity rate—are consistent with previously reported individual-level correlates of cirrhosis and pneumonia-influenza outcomes. However, it is critical to emphasize that the following mechanistic interpretations, drawn from prior clinical and biological studies, are not validated by our ecological data and are offered solely as contextual background for observed population-level associations. Among these, obesity has been associated with worsening liver fibrosis progression and elevated lung infection risk through metabolic inflammation and immune dysregulation in clinical cohorts (21–23). The poorer health status represented by a higher number of physically unhealthy days has been linked to factors such as a relatively low ratio of social services and public health expenditures to healthcare expenditures, and unhealthy lifestyles (24, 25). These social and behavioral factors may parallel unfavorable disease outcomes through dual pathways: on one hand, they may coincide with incomplete long-term management of chronic diseases such as liver fibrosis-cirrhosis; on the other hand, they may align with delayed care-seeking for acute infections, a pattern associated with higher mortality rates in prior studies (26). Furthermore, a higher disconnected youth rate correlates with elevated local prevalence of violence, smoking, drinking, and marijuana use in the area, with these patterns accompanied by poorer population health metrics (27). These factors are intertwined with prevalent social determinants such as poverty, low education levels, and weak health infrastructure, potentially forming a “social-behavioral-clinical” risk chain at the county level. Collectively, these findings provide observational context relevant to targeted prevention and control strategies addressing the comorbidity, while recognizing that individual-level causal pathways cannot be inferred from our county-level aggregate data.

This county-level ecological analysis integrates spatial epidemiological methods with county clustering to examine associations between geographic features, county subgroup profiles, and aggregate comorbidity mortality. To overcome the limitations of single-factor investigations, we constructed a multi-dimensional analytical workflow combining PCA-HCPC clustering, LASSO regression for covariate screening, and generalized linear models for association testing—establishing a sequential framework of “dimensionality reduction, county grouping, and association verification” that enhances the robustness of ecological findings. The phenotypic classification developed in this study is intended to offer a practical, operationally actionable framework for county-level health planning, rather than a purely academic exercise. We propose the following concrete translational pathways. First, for county health departments and state-level public health agencies, the three phenotype assignments can serve as a risk-stratification tool for resource allocation. Counties classified as Phenotype 2—characterized by high physically unhealthy days, high obesity, and low primary care access—could be prioritized for integrated “comorbidity-preparedness” funding streams that jointly support chronic liver disease management (e.g., hepatitis C screening, alcohol harm reduction) and respiratory infection prevention (e.g., targeted influenza vaccination campaigns, pneumonia surveillance). Second, for healthcare systems and community health centers operating in Phenotype 2 counties, the findings suggest the need for combined intervention packages rather than single-domain programs. For instance, embedding influenza vaccination and pneumonia education within existing chronic disease management clinics, or co-locating obesity counseling with respiratory infection screening, could address the multidomain risk configuration more efficiently than separate, siloed initiatives. Third, for local surveillance and early warning systems, the county-level CHR indicators used in our clustering—which are updated annually and publicly available—can be monitored as leading ecological proxies. A county approaching Phenotype 2 thresholds on key dimensions (e.g., physically unhealthy days > 4.0 days, disconnected youth > 9%, adult obesity > 34%) could trigger pre-emptive public health alerts and resource pre-positioning ahead of the influenza season, without waiting for lagged mortality data. We emphasize that these proposed applications are observational and hypothesis-generating; they require prospective implementation and evaluation to validate their effectiveness in reducing comorbidity mortality. The phenotype classification is not intended to replace individual-level clinical risk assessment but to complement it by providing a macro-level framework for population health management.

However, our study has several limitations. First, our Joinpoint trend analysis identified only a single turning point (2011) and did not include a leave-one-period-out sensitivity analysis excluding the pandemic years (2020–2023). While we observed a significant upward trend from 2011 to 2023 (APC = 6.28%), we cannot definitively determine whether this sustained increase was primarily driven by long-term secular trends (e.g., rising obesity, opioid epidemic), exacerbated by the severe 2017–2018 influenza season, or predominantly attributable to the COVID-19 mortality shock. The concurrent and overlapping nature of these temporal exposures prevents us from statistically isolating the independent effect of each event. Future studies with longer post-pandemic follow-up or those employing time-series intervention analyses (e.g., interrupted time series with multiple intervention points) could help clarify the relative contributions of these overlapping temporal factors. Second, our study has selection bias inherent to CDC WONDER data rules. The database suppresses county mortality data with fewer than 20 annual deaths and labels these values unreliable. We only retained 435 counties with complete 2018–2023 mortality data, systematically excluding small rural counties that often face scarce medical resources, weak liver disease management and concentrated health risks, and carry heavy cirrhosis-influenza-pneumonia mortality burdens. Table 1 shows median county populations between 132,064 and 678,701, proving our sample mainly covers medium and large counties without remote rural areas. This bias may underestimate the impacts of poor physical health, disconnected youth and obesity on rural comorbidity mortality. Additionally, our identified spatial clusters, three risk phenotypes and temporal trends only apply to populous counties and cannot be generalized nationwide. Future work can use raw individual death records to include small rural counties and perform stratified analyses by population size and urban–rural classification to clarify distinct mortality drivers. Third, this ecological analysis at the county level cannot infer individual-level causal relationships, which creates risks of ecological fallacy. All statistical associations observed between county-level indicators and comorbidity mortality reflect population-level aggregated patterns rather than biological or behavioral causal effects in single individuals. The correlated area-level markers (poor physical health status, high disconnected youth rate, and elevated adult obesity prevalence) identified in our models do not confirm that these individual conditions directly increase mortality risk at a personal level. Mechanistic causal pathways linking obesity, chronic physical illness and respiratory infection mortality cited in the Discussion are derived from prior individual clinical studies, and cannot be validated by our cross-county aggregate data alone. Fourth, the County Health Rankings (CHR) 2020 variables used in this study represent a single cross-sectional snapshot, with different measures drawn from various prior years. This static exposure cannot establish temporal precedence relative to the multi-year mortality outcome (2018–2023) or the longer-term trend (1999–2023). The absence of temporality precludes any causal interpretation of the observed associations. Our findings should therefore be understood as ecological correlations between county-level characteristics measured around 2020 and aggregate mortality rates in the subsequent period, rather than as evidence that these characteristics ‘caused’ or ‘drove’ the observed mortality patterns. Future studies could leverage multiple years of CHR data (available annually since 2010) to construct time-varying exposure models that better address the temporality requirement. Fifth, our study used annual mean PM_2.5_ concentration as an environmental covariate, as the CHR database only reports PM_2.5_ as an annual average aggregated by county year and does not provide seasonal or monthly breakdowns. For respiratory infection outcomes such as pneumonia and influenza, exposure during the influenza season may be more etiologically relevant than annual averages. However, due to data availability constraints in the CHR dataset, we were unable to incorporate season-specific PM_2.5_ estimates. Future studies integrating satellite-derived PM_2.5_ data at monthly or weekly resolutions could provide more temporally refined exposure assessments and better characterize the acute effects of air pollution on comorbidity mortality. Additionally, as the conclusions are based on U.S. data, the high-risk phenotype characteristics identified here require contextual adaptation when applied to other settings. In the future, community intervention trials could be conducted in the high-risk areas identified in this study to validate and optimize the effectiveness of targeted strategies.

## 5 Conclusion

This county-level ecological analysis demonstrates that aggregate county mortality from concurrent liver fibrosis-cirrhosis and pneumonia-influenza across the U.S. rose in distinct phases between 1999 and 2023, with a marked upward ecological shift after 2011. In-depth analysis identified three key population-level factors associated with high comorbidity mortality: average number of physically unhealthy days, disconnected youth rate, and adults with obesity rate. The observed ecological associations deliver population-scale observational reference for developing geographically tailored public health plans and designing county-based integrated surveillance frameworks.

## Data Availability

The raw data supporting the conclusions of this article will be made available by the authors, without undue reservation.
