# Supplementary material for: Trends and factors associated with liver fibrosis-cirrhosis complicated with pneumonia-influenza mortality in U.S. Counties: a CDC WONDER and CHR database analysis
Source: Front Public Health. 2026 Aug 12;14:1884089. doi: 10.3389/fpubh.2026.1884089 (PMC13506777; doi:10.3389/fpubh.2026.1884089)
Supplement: Supplementary file 1 [file Table_1.docx]

Supplementary Material

## Supplementary Table 1 Baseline characteristics comparison between included and excluded counties

| Variable | Overall | Included | Excluded | p-value^2^ |
| --- | --- | --- | --- | --- |
|  | N = 3,143^1^ | N = 435^1^ | N = 2,708^1^ |  |
| %.Fair.or.Poor.Health | 17.92 (4.73) | 17.65 (4.38) | 17.96 (4.78) | 0.168 |
| Average.Number.of.Physically.Unhealthy.Days | 3.99 (0.70) | 3.95 (0.64) | 4.00 (0.71) | 0.142 |
| Average.Number.of.Mentally.Unhealthy.Days | 4.17 (0.60) | 4.20 (0.55) | 4.16 (0.61) | 0.166 |
| %.Low.Birthweight | 8.15 (2.06) | 8.33 (1.46) | 8.12 (2.14) | 0.009 |
| %.Smokers | 17.45 (3.58) | 16.43 (3.64) | 17.62 (3.55) | <0.001 |
| %.Adults.with.Obesity | 32.90 (5.45) | 30.87 (5.36) | 33.23 (5.40) | <0.001 |
| Food.Environment.Index | 7.46 (1.13) | 7.59 (0.94) | 7.43 (1.16) | 0.002 |
| %.Physically.Inactive | 27.42 (5.71) | 24.79 (5.47) | 27.85 (5.63) | <0.001 |
| %.With.Access.to.Exercise.Opportunities | 62.83 (23.37) | 81.09 (15.68) | 59.89 (23.07) | <0.001 |
| %.Excessive.Drinking | 17.52 (3.17) | 18.17 (2.90) | 17.41 (3.19) | <0.001 |
| %.Driving.Deaths.with.Alcohol.Involvement | 28.30 (14.95) | 28.12 (7.51) | 28.33 (15.84) | 0.652 |
| Chlamydia.Rate | 401.07 (283.02) | 507.30 (239.94) | 383.03 (285.84) | <0.001 |
| Teen.Birth.Rate | 29.85 (14.14) | 25.71 (12.47) | 30.56 (14.29) | <0.001 |
| %.Uninsured | 11.47 (5.15) | 10.49 (4.87) | 11.63 (5.18) | <0.001 |
| Primary.Care.Physicians.Rate | 54.51 (35.28) | 73.48 (28.49) | 51.29 (35.31) | <0.001 |
| Dentist.Rate | 45.81 (31.56) | 65.48 (25.40) | 42.55 (31.31) | <0.001 |
| Mental.Health.Provider.Rate | 160.80 (173.38) | 247.02 (183.25) | 145.68 (167.11) | <0.001 |
| Preventable.Hospitalization.Rate | 4,858.50 (1,840.84) | 4,826.37 (1,583.09) | 4,863.74 (1,879.71) | 0.658 |
| %.With.Annual.Mammogram | 40.67 (7.66) | 41.56 (5.98) | 40.53 (7.89) | 0.002 |
| %.Vaccinated | 41.76 (9.76) | 46.74 (6.37) | 40.95 (9.97) | <0.001 |
| High.School.Graduation.Rate | 88.76 (7.16) | 86.58 (6.23) | 89.13 (7.24) | <0.001 |
| %.Some.College | 57.90 (11.82) | 63.80 (9.98) | 56.95 (11.82) | <0.001 |
| %.Unemployed | 4.13 (1.47) | 4.18 (1.55) | 4.12 (1.46) | 0.409 |
| %.Children.in.Poverty | 21.14 (8.88) | 19.16 (8.18) | 21.46 (8.95) | <0.001 |
| Income.Ratio | 4.51 (0.76) | 4.70 (0.74) | 4.48 (0.76) | <0.001 |
| %.Single-Parent.Households | 32.35 (10.64) | 33.84 (8.25) | 32.11 (10.96) | <0.001 |
| Social.Association.Rate | 11.67 (5.91) | 9.25 (3.13) | 12.06 (6.15) | <0.001 |
| Violent.Crime.Rate | 251.99 (192.48) | 380.19 (241.03) | 229.99 (173.65) | <0.001 |
| Average.Daily.PM2.5 | 9.02 (1.97) | 9.92 (1.84) | 8.88 (1.95) | <0.001 |
| %.Severe.Housing.Problems | 13.84 (4.43) | 16.94 (4.64) | 13.34 (4.19) | <0.001 |
| %.Drive.Alone.to.Work | 79.67 (7.56) | 79.05 (8.93) | 79.77 (7.32) | 0.109 |
| %.Long.Commute.-.Drives.Alone | 31.47 (12.51) | 34.05 (11.00) | 31.06 (12.69) | <0.001 |
| %.Frequent.Physical.Distress | 12.12 (2.34) | 11.96 (2.17) | 12.15 (2.37) | 0.097 |
| %.Frequent.Mental.Distress | 13.00 (1.99) | 12.94 (1.80) | 13.00 (2.01) | 0.5 |
| %.Adults.with.Diabetes | 12.17 (4.06) | 11.15 (2.98) | 12.34 (4.19) | <0.001 |
| HIV.Prevalence.Rate | 183.96 (205.23) | 310.12 (328.46) | 158.83 (158.96) | <0.001 |
| %.Food.Insecure | 13.29 (3.96) | 13.14 (3.61) | 13.31 (4.01) | 0.352 |
| %.Limited.Access.to.Healthy.Foods | 8.62 (8.31) | 7.41 (4.86) | 8.81 (8.72) | <0.001 |
| %.Insufficient.Sleep | 33.04 (4.16) | 34.47 (3.56) | 32.81 (4.21) | <0.001 |
| %.&#10;Uninsured.adults | 13.57 (6.23) | 12.72 (6.12) | 13.71 (6.24) | 0.002 |
| %.&#10;Uninsured.children | 6.27 (3.67) | 4.95 (2.72) | 6.48 (3.76) | <0.001 |
| Other.Primary.Care.Provider.Rate | 84.98 (62.46) | 106.23 (52.93) | 81.55 (63.21) | <0.001 |
| Average.Grade.Performance(Reading.scores) | 3.04 (0.28) | 3.08 (0.24) | 3.04 (0.29) | 0.005 |
| Average.Grade.Performance(Math.scores) | 3.01 (0.32) | 3.04 (0.28) | 3.00 (0.32) | 0.032 |
| Median.Household.Income | 52,807.54 (13,874.79) | 61,538.04 (17,792.75) | 51,406.78 (12,590.32) | <0.001 |
| %.Enrolled.in.Free.or.Reduced.Lunch | 53.88 (18.61) | 52.82 (16.04) | 54.04 (18.98) | 0.166 |
| Segregation.index(Residential.segregation.-.Black/White) | 45.18 (16.42) | 50.64 (11.84) | 43.75 (17.14) | <0.001 |
| Segregation.Index(Residential.segregation.-.non-White/White) | 30.81 (13.19) | 37.14 (11.57) | 29.65 (13.14) | <0.001 |
| Firearm.Fatalities.Rate | 15.55 (6.97) | 12.92 (5.92) | 16.19 (7.06) | <0.001 |
| Juvenile.Arrest.Rate | 26.21 (18.74) | 22.68 (13.88) | 26.95 (19.53) | <0.001 |
| Average.Traffic.Volume.per.Meter.of.Major.Roadways | 128.84 (274.09) | 380.86 (562.39) | 88.42 (157.25) | <0.001 |
| %.Homeowners | 71.44 (8.27) | 65.82 (9.16) | 72.34 (7.75) | <0.001 |
| %.Severe.Housing.Cost.Burden | 11.26 (3.61) | 14.06 (3.64) | 10.81 (3.39) | <0.001 |
| %.less.than.18.years.of.age | 22.06 (3.45) | 22.41 (3.13) | 22.00 (3.50) | 0.013 |
| %.65.and.over | 19.28 (4.70) | 16.65 (4.69) | 19.70 (4.56) | <0.001 |
| %.Black | 9.00 (14.30) | 11.66 (12.26) | 8.57 (14.56) | <0.001 |
| %.American.Indian.&.Alaska.Native | 2.30 (7.39) | 1.71 (6.21) | 2.39 (7.56) | 0.04 |
| %.Asian | 1.57 (2.95) | 4.31 (5.45) | 1.13 (1.99) | <0.001 |
| %.Native.Hawaiian/Other.Pacific.Islander | 0.14 (0.97) | 0.23 (0.81) | 0.12 (0.99) | 0.01 |
| %.Hispanic | 9.66 (13.84) | 16.16 (17.41) | 8.61 (12.88) | <0.001 |
| %.Non-Hispanic.White | 76.04 (20.12) | 64.41 (20.91) | 77.91 (19.35) | <0.001 |
| %.Not.Proficient.in.English | 1.67 (2.83) | 3.45 (4.01) | 1.38 (2.47) | <0.001 |
| %.Female | 49.89 (2.28) | 50.79 (1.48) | 49.74 (2.36) | <0.001 |
| %.Rural | 58.58 (31.48) | 19.91 (19.55) | 64.80 (28.46) | <0.001 |
| Note: 1 Mean (SD) | | | | |
| 2 Welch Two Sample t-test | | | | |
